# Palbociclib to reverse endocrine resistance in breast cancer: a TREnd in the right direction?

**DOI:** 10.18632/oncotarget.26133

**Published:** 2018-09-25

**Authors:** Amelia McCartney, Angelo Di Leo, Luca Malorni

**Affiliations:** Luca Malorni: Sandro Pitigliani Medical Oncology Department, Hospital of Prato, Istituto Toscano Tumori, Prato, Italy; Translational Research Unit, Hospital of Prato, Istituto Toscano Tumori, Prato, Italy

**Keywords:** endocrine resistance, CDK4/6 inhibitors, breast cancer, metastatic, palbociclib

The entry of CDK4/6 inhibitors to the management of hormone receptor-positive, HER2-negative metastatic breast cancer (BC) has changed clinical practice, in many patients delaying the need to initiate potentially more toxic treatment lines such as cytotoxic chemotherapy. Landmark phase 3 trials testing the efficacy and tolerability of three different CDK4/6 inhibitors, palbociclib [1, 2], ribociclib [3, 4] and abemaciclib [5, 6] have collectively and consistently shown an approximate doubling of progression-free survival (PFS) when CDK4/6 inhibitors are given in combination with endocrine therapy (ET), either as initial treatment for metastatic disease, or in subsequent lines following previous disease progression. The majority of BCs positively express the oestrogen receptor (ER), and ET forms an essential, principally well-tolerated backbone to the management of metastatic hormone receptor-positive BC. However, resistance to ET over time is considered inevitable, with the consequent loss of disease control necessitating more onerous treatment regimens. As such, extending or improving the activity of ET is of utmost clinical relevance and importance.

The TREnd (To Reverse ENDocrine resistance) trial was a phase 2, open label, multicentre randomised study of palbociclib given as monotherapy, or in combination with the endocrine therapy upon which the patient had progressed in the previous line [7]. Women were eligible if they were post-menopausal, with advanced or metastatic ER-positive, HER2-negative BC, with a history of having had disease progression during the previous-line (1^st^ or 2^nd^) ET given for advanced breast cancer. At the time TREnd was designed and initiated in eight different cancer centres in Italy, there were no published phase 3 data on CDK4/6 inhibitors, and as such, the optimum treatment regimen and clinical setting was not yet established. The endpoints of TREnd aimed to assess the activity, safety and efficacy of palbociclib when given as monotherapy, as well as to investigate whether palbociclib had clinical capacity to reverse established endocrine resistance, as had been shown previously in pre-clinical models [8].

The primary endpoint of TREnd was met, and both arms were declared active based on study assumptions, with the clinical benefit rate being 54% (95% CI 41.5-63.7) in the combination arm, and 60% (95% CI 47.8-72.9) in the monotherapy arm. The median PFS was 10.8 months (95% CI 5.6-12.7) in the combination group, and 6.5 months (95% CI 5.4-8.5) in the monotherapy arm (HR = 0.69, 95% CI 0.4-1.1, exploratory P-value = 0.12). Previously, palbociclib monotherapy was shown to be active in heavily pre-treated patients [9], and accordingly, TREnd added to this data in a less pre-treated population. However, during the course of TREnd, the intervening publication of seminal CDK4/6 trials resulted in the widespread adoption of combining CDK4/6 agents with ET, essentially superseding CDK4/6 monotherapy as a treatment option, rendering the primary endpoint of TREnd somewhat clinically redundant, despite its affirmative findings. Nevertheless, indirect comparisons of outcome between trials suggest the median PFS observed in the moderately pre-treated population of TREnd is akin to that observed in similar populations that received second or third-line chemotherapy or targeted agents in other trials [10, 11]. Succinctly, single-agent palbociclib may still offer similar prospective survival benefits as other, potentially more toxic, regimens.

Of more contemporaneous relevance were the secondary endpoint findings pertaining to the reversal of endocrine resistance. Exploratory analyses were performed to determine if one group of patients received a larger benefit from either of the study arms. Whilst the clinical benefit rate was found to be similar between the two arms overall (54% *versus* 60%), the median duration of clinical benefit was 11.5 months (95% CI 8.5-17.8) in the combination arm, and 6 months (95% CI 3.9-10.8) in the monotherapy group (HR=0.35, 95% CI 0.18-0.70, exploratory *P*-value = 0.0021). This significantly longer duration of benefit in the combination arm may be attributed to additional therapeutic action of ET, following the reversal of prior resistance by palbociclib. Similarly, PFS advantage for combination therapy was seen only in the subgroup of patients who received prior ET in excess of six months (HR 0.53, 95% CI 0.3-4.0, exploratory P-value 0.02), but not in the subgroup who received prior endocrine therapy for less than six months.

These groups cannot be viewed as classically analogous models of primary and secondary endocrine resistance, as the stratification of duration of prior ET (less than or more than six months) was arbitrary and not based on formal consensus-approved definitions of endocrine resistance [12]. However, these findings still present the intriguing hypothesis that in patients with a previous durable response to endocrine therapy, some synergism may still remain between palbociclib and ET, and resistance may be at least partially reversed by combining the two agents. Conversely, in patients with a previous short-lived response to ET, there seemed to be little, if any, benefit in continuing the preceding line of ET in combination with palbociclib – perhaps suggesting the presence of a significantly different mechanism of endocrine resistance. These findings are particularly interesting when viewed in context of PALOMA-3 [2], wherein a strategy of switching to a new endocrine therapy and adding palbociclib was adopted following previous disease progression on endocrine therapy, resulting in a median PFS of 9.5 months with combined palbociclib and fulvestrant. Additionally, a recent single-centre study of 60 heavily pre-treated patients who received fulvestrant and palbociclib for advanced disease reported a median PFS of 5.8 months, whilst intriguingly, the PFS in those patients who had received fulvestrant in a past line of therapy (*n* = 28; 46.7%) was 6.4 months [13] – perhaps signalling additional evidence of endocrine resistance reversal by concomitant CDK4/6 therapy. Translational studies on TREnd are in progress, and may provide some correlation with regards to associated biomarkers of resistance. In future trials incorporating patients with ET-resistant disease, further pursuit of a strategy to prolong the action of the existing ET beyond progression with the addition of CDK4/6 inhibitor therapy may be of interest.
